# Efficiency of two-phase methods with focus on a planned population-based case-control study on air pollution and stroke

**DOI:** 10.1186/1476-069X-6-34

**Published:** 2007-11-07

**Authors:** Anna Oudin, Jonas Björk, Ulf Strömberg

**Affiliations:** 1 Department of Occupational and Environmental Medicine, Lund University Hospital, Lund, Sweden; 2 Competence Centre for Clinical Research, Lund University Hospital, Lund, Sweden

## Abstract

**Background:**

We plan to conduct a case-control study to investigate whether exposure to nitrogen dioxide (NO_2_) increases the risk of stroke. In case-control studies, selective participation can lead to bias and loss of efficiency. A two-phase design can reduce bias and improve efficiency by combining information on the non-participating subjects with information from the participating subjects. In our planned study, we will have access to individual disease status and data on NO_2 _exposure on group (area) level for a large population sample of Scania, southern Sweden. A smaller sub-sample will be selected to the second phase for individual-level assessment on exposure and covariables. In this paper, we simulate a case-control study based on our planned study. We develop a two-phase method for this study and compare the performance of our method with the performance of other two-phase methods.

**Methods:**

A two-phase case-control study was simulated with a varying number of first- and second-phase subjects. Estimation methods: *Method 1*: Effect estimation with second-phase data only. *Method 2*: Effect estimation by adjusting the first-phase estimate with the difference between the adjusted and unadjusted second-phase estimate. The first-phase estimate is based on individual disease status and residential address for all study subjects that are linked to register data on NO_2_-exposure for each geographical area. *Method 3*: Effect estimation by using the expectation-maximization (EM) algorithm without taking area-level register data on exposure into account. *Method 4*: Effect estimation by using the EM algorithm and incorporating group-level register data on NO_2_-exposure.

**Results:**

The simulated scenarios were such that, unbiased or marginally biased (< 7%) odds ratio (OR) estimates were obtained with all methods. The efficiencies of method 4, are generally higher than those of methods 1 and 2. The standard errors in method 4 decreased further when the case/control ratio is above one in the second phase. For all methods, the standard errors do not become substantially reduced when the number of first-phase controls is increased.

**Conclusion:**

In the setting described here, method 4 had the best performance in order to improve efficiency, while adjusting for varying participation rates across areas.

## Background

We consider a planned study on possible long-term effects of exposure to air pollution on the incidence of stroke in Scania, a region in the southernmost part of Sweden. There have been several previous studies investigating the association between exposure to air pollution and the risk of developing stroke, with both positive and negative findings [1-4]. Early studies reported adverse health effects of high levels of air pollution [5].

Exposure to nitrogen dioxide (NO_2_) has documented momentary and chronic health effects [6]. There have been several time-series studies using peak values or daily mean concentrations of NO_2 _as indicator of exposure, where short-term associations with stroke admissions to hospitals have been shown [3,7,8]. Proximity to roads has been used as an indicator of air pollution in general, revealing an association with long-term incidence of stroke [9].

Increased cardio-respiratory mortality has been reported as an effect of chronic exposure to outdoor air pollution but without specific reports on stroke [10-12]. Maheswaran et al. [13] showed an association between levels of air pollution and incidence of stroke in a area-level geographical study, where potential confounders such as smoking and socio-economic status were adjusted for at the area-level. However, adjusting for confounding on group/area-level cannot generally replace adjustments on the individual-level [14].

We have access to area-level concentrations of air pollution from dispersion modelling [15], and access to individual-level data on stroke occurrence together with age, sex, and current residential area for the entire population in Scania. For about half of the population, we also have access to the geographical coordinates of each persons' past residences from 1984 and onwards. Furthermore, we will have access to individual data on exposure to air pollution as well as on potential confounding factors such as smoking for a subsample. Our data make it possible to investigate long-term effect of air pollution and possible synergistic effects of age, sex, and smoking.

A two-phase design is a design where some variables, here called first-phase variables, are determined for all study subjects, for example from registers, while a sub-sample of subjects, here called second-phase subjects, is selected for additional data collection [16]. Missing-data methods can be applied to estimate the values on exposure and covariables in the subjects not selected into the second phase [16]. We provide a more detailed description of the two-phase concept and a brief history of the development of two-phase methods in the additional file 1.

In this paper, we simulate data from a two-phase design that resemble our planned case-control study on the long-term effects of air pollution on stroke risk. Available data in the first-phase are individual disease status and residential address for all study subjects that are linked to register data on NO_2_-exposure for each geographical area. The register data are formulated as probabilities of low, medium, and high exposure. Supplementary individual-level data on exposure and confounders are assessed for a sub-sample in the second phase. Strömberg and Björk [17] have considered a similar setting and we now extend their work in two important ways:

1) we allow for a trichotomous exposure (with straightforward generalizations to multiple exposure categories) on the individual level;

2) we allow for collection of data on confounders, together with individual-level exposure data, in the second phase.

We also compare the efficiency of the effect estimates of typical two-phase methods, calculated relative to the ideal (but unrealistic) situation with second-phase data on all study subjects being available. In particular, we want to compare the efficiency of two-phase methods using and not using register area-level exposure data available for the first-phase subjects.

## Methods

### General study scenario

In our planned study, we use exposure to NO_2 _as a marker of air pollution. The first-phase data on residential addresses of the study subjects that can be linked to a Geographical Information Systems (GIS) that provides exposure data on the area level [15]. The area-level exposure distribution in our setting is characterized through probabilities of exposure at low, medium, and high level rather than through area-level mean values. Each study subject is therefore assigned three exposure probabilities that sum up to one based on the residential address – to resemble empirical data obtained with GIS technique (table 2). In the second phase, the individual level of NO_2 _(low, medium, or high) is assessed using interview information, for example questions about time spent out of doors, location, and the usual route to and from work. We assume confounding but no effect modification by smoking. No external information about smoking is available, but is obtained individually in the second-phase interviews together with additional data collection used to assess the exposure to NO_2_.

**Table 2:** Hypothetical population distribution in Scania

		Group-level exposure probabilities for 12 areas* within the categories Low Medium and High (%)												Smoking prevalence within exposure categories † (%)
	Area*	1	2	3	4	5	6	7	8	9	10	11	12	
	Low**	92	89	88	85	77	60	40	35	32	20	20	16.5	5
Exposure category	Medium**	7	10	60	14	20	37	30	60	42	75	18	18.5	25
	High**	1	1	5	1	3	3	30	5	26	5	62	65	40
First-phase control distribution (%)		2	9.5	3	7	0.5	4	5	12	15	6	1	35	

* The areas represent 12 municipalities in Scania, Southern Sweden.

** Overall exposure prevalence to the categories Low, Medium and High was 40%, 30% and 30%, respectively.

† Overall smoking prevalence in the population is 22%.

### Modelling framework

A logistic, and thus multiplicative, model is used on the individual level:

logit(*p*) = (*β*_0 _+ *β*_1_·*x*_1 _+ *β*_2_·*x*_2 _+ *β*_3_·*x*_3_)

where *p *denotes probability of being a case. Two binary indicator variables characterize the NO_2_-exposure at three different levels: *x*_1 _= 1 if medium exposure and *x*_2 _= 1 if high exposure. Smoking status is binary and indicated by *x*_3_(*x*_3 _= 1 if smoker). The baseline risk is denoted by *β*_0_, *β*_0_, *β*_1 _and *β*_2_, are the effects of exposure and *β*_3 _is the effect of smoking.

We assume 12 different geographical areas (*k *= 1, 2, ..., 12). At the ecological level, the exposure category distribution in each area is characterized by three exposure probabilities, viz. the proportion of subjects classified in the low (*X*_0*k*_), medium (*X*_1*k*_) and high (*X*_2*k*_) categories, respectively, where *X*_0*k *_= 1 - *X*_1*k *_- *X*_2*k*_. Individual disease status and residential area are known for all study, whereas smoking status is unknown. We assume a linear odds model for the ecological association across the geographical areas [18], where each area is characterized by (*X*_1*k*_) and (*X*_2*k*_):

Θ_k _= *e*^*α*^·(1 + *λ*_1_·*X*_1*k *_+ *λ*_2_·*X*_2*k*_)

where Θ_k _is the expected disease odds for a subject living in area *k*, *e*^*α *^is the baseline disease odds (i.e., when the probabilities of medium and high exposure both are zero), 1 + λ_1 _is disease odds ratio (OR) for medium vs. low exposure to NO_2_, and 1 + λ_1 _is disease OR for high vs. low exposure to NO_2_. Note that we expect that exp(*β*_1_) = 1 + λ_1 _and exp(*β*_2_) = 1 + λ_2 _only when there is no confounding from smoking on the ecological association and no other ecological bias is present.

In practice, data can be expected to be spatially correlated within geographical areas and, therefore, a random effect model is often applied [19]. The individual-level model (equation 1) can be extended to include random effects by replacing the fixed baseline risk *β*_0 _with a random area-dependent baseline risk *β*_0*k *_[20]. Here, for simplicity, no such spatial correlation is assumed.

### Simulations

We designed the simulation scenarios with consideration being to our planned case-control study in Scania, Sweden. Stroke is a relatively rare disease with an incidence of approximately 2 cases per 1,000 person-years in the total population [21]. The planned recruitment period for this study is one year, with inclusion of all incident cases in the 12 largest municipalities (areas) in Scania (total population of approximately 800,000), and we can expect approximately 1,600 new cases in the region during that year. The first-phase controls are sampled among people who are disease-free at the beginning of the recruitment period [22].

We assume that no selection bias arises from the recruitment of the first-phase cases and controls. Due to the register-based recruitment in this study and the quality of the population registers available in Sweden, this assumption is not hazardous.

Three true ORs were assumed: 1.5 (medium vs. low level of NO_2 _exposure), 3 (high vs. low level of NO_2 _exposure) and 2 (smoker vs. non-smoker), corresponding to *β*_1 _= log(1.5), *β*_2 _= log(3) and *β*_3 _= log(2) in equation 1.

In the three scenarios in table 3, the number of second-phase cases and controls are fixed (at 300 each), whereas the number of first-phase subjects varies. In table 4, the number of first-phase cases and controls are held fixed (at 1,200 each). The total number of second-phase subjects in table 4 is 600 for all three scenarios, but the distributions of cases and controls vary. For all scenarios, 1,000 replications were carried out.

**Table 3:** Simulation results based on 1,000 replications

		Scenario 1 Number of first-phase subjects: 400 cases 1,200 controls			Scenario 2 Number of first-phase subjects: 1,200 cases 1,200 controls			Scenario 3 Number of first- phase subjects: 400 cases 12,000 controls		
	True OR††	OR*	SD*	Ef**	OR*	SD*	Ef**	OR*	SD*	Ef**

Individual-level information on all subjects †	1.50	1.49	0.18	100	1.50	0.12	100	1.50	0.16	100
	3.00	3.00	0.17	100	3.00	0.11	100	3.00	0.14	100
Method 1‡	1.50	1.48	0.25	53	1.50	0.24	25	1.50	0.25	43
	3.00	3.01	0.24	51	2.98	0.23	23	2.99	0.23	40
Method 2‡	1.50	1.60	0.32	29	1.56	0.24	25	1.59	0.29	28
	3.00	3.05	0.27	39	3.01	0.18	39	3.04	0.24	37
Method 3‡	1.50	1.49	0.24	58	1.50	0.22	29	1.50	0.24	46
	3.00	3.00	0.22	59	2.98	0.21	29	3.00	0.21	47
Method 4‡	1.50	1.49	0.19	81	1.50	0.20	37	1.51	0.19	68
	3.00	2.99	0.16	78	3.01	0.17	44	3.02	0.18	66

* Geometric mean of the OR estimates and the empiric standard deviation of the ln(OR) estimates.

** Efficiency of the ln(OR) estimates. eff_1 _= (var(ln(OR_1_)) + (ln(true OR_1_))-ln(OR_1_)))/(var(ln(OR_ref_)) + (ln(true OR_ref_))-ln(OR_ref_))) where OR_ref _is the estimate in the ideal scenario. Efficiencies calculated when varying the number of first-phase subjects. The number of second-phase cases and controls are held fixed at 300 cases and 300 controls.

† Ideal scenario.

‡ Methods 1–4 are further described in the Methods section and in Table 1.

†† A confounder with OR = 2 is introduced and a positive bias-effect of 20% for OR = 1.50 and 33% for OR = 3.00

**Table 4:** Simulation results based on 1,000 replications

		Scenario 2.1*** Number of second-phase subjects: 300 cases 300 controls			Scenario 2.2 Number of second-phase subjects: 400 cases 200 controls			Scenario 2.3 Number of second- phase subjects: 200 cases 400 controls		
	True OR††	OR*	SD*	Ef**	OR*	SD*	Ef**	OR*	SD*	Ef**

Individual-level information on all subjects †	1.50	1.50	0.12	100	1.49	0.12	100	1.50	0.12	100
	3.00	3.00	0.11	100	3.02	0.12	100	3.02	0.12	100
Method 3‡	1.50	1.50	0.22	29	1.49	0.23	27	1.52	0.22	30
	3.00	2.98	0.21	29	2.97	0.21	30	3.05	0.20	35
Method 4‡	1.50	1.50	0.20	37	1.51	0.18	46	1.51	0.21	34
	3.00	3.01	0.17	44	3.02	0.16	51	3.04	0.17	46

* Geometric mean of the OR estimates and the empiric standard deviation of the ln(OR) estimates.

** Efficiency of the ln(OR) estimates. eff_1 _= (var(ln(OR_1_)) + (ln(true OR_1_))-ln(OR_1_)))/(var(ln(OR_ref_)) + (ln(true OR_ref_))-ln(OR_ref_))) where OR_ref _is the estimate in the ideal scenario. Efficiencies calculated when varying the number of second-phase cases and controls. The number of first-phase cases and controls are held fixed at 1,200 cases and 1,200 controls.

*** These results are also presented in table 3 (scenario 2).

† Ideal scenario.

‡ Methods 3–4 are further described in the Methods section and in Table 1.

†† A confounder with OR = 2 is introduced and a positive bias-effect of 20% for OR = 1.50 and 33% for OR = 3.00

In each replication the subjects were selected randomly, so that the distributions of the exposure and co-variables among the second-phase cases and controls would reflect those distributions in the population. The overall prevalence of smoking was set to be 22% in accordance to official Swedish statistics [23]. Smoking prevalence in the different exposure categories was assumed according to table 2, but there was no variation in smoking prevalence in the NO_2_-exposure categories between areas. Thus, the exposure-disease association is confounded by smoking at the individual level [24]. The effect estimates and empiric standard deviations were calculated for all four methods as well as the efficiencies. The efficiencies were calculated as the ratio of the mean square error of the ideal scenario (individual-level information on all subjects) and the mean square error provided by the estimation method at issue.

### Estimation methods

Table 1 outlines how data are used in the estimation methods we examine. We consider the following methods:

**Table 1:** Overview of the four combinations of first- and second-phase data evaluated in this paper

	First-phase registry data			Second-phase interview and measurement data	
Type of design	Disease status	Residential area	Exposure	Exposure	Covariates

1. No first-phase exposure data (method 1)	Individual	Individual	-	Individual	Individual
2. First- and second-phase exposure data (method 2 and method 4)	Individual	Individual	Area	Individual	Individual
3. No first-phase exposure data (method 3)	Individual	Individual	-	Individual	Individual

#### Method 1

We estimate the parameters of the individual-level model (equation 1) from the second-phase data only. Hence, method 1 is not a two-phase method but it is often used in practice among epidemiologists, for example due to non-response in case-control studies.

#### Method 2

The first-phase effect estimates for this method are calculated using individual-level disease status and area-level exposure data; that is, a partially ecologic estimation procedure based on equation 2 [19]. We then adjust the first-phase effect estimates with the difference between the corresponding smoking-adjusted and unadjusted second-phase estimates obtained from the individual-level logistic model (equation 1; see also additional file 2[25]).

#### Method 3

We estimate the parameters of the individual-level model based on both first- and second-phase subjects by using missing-data methods based on the EM algorithm (additional file 3). Briefly, in the initial expectation (E) step, the individual-level data obtained in the second phase on air pollution exposure and smoking provide estimates of exposure and smoking effects. Consequently, we can estimate the expected frequency distribution with respect to the exposure and smoking categories across the areas for the non-participating (i.e., only-first-phase) cases and controls, respectively. In the maximization (M) step, we obtain new effect estimates based on the total (observed + expected) numbers of cases and controls within each category of exposure level and smoking status. The E- and M-steps are repeated until the effect estimates converge.

#### Method 4

We use the EM algorithm to obtain effect estimates as if the total frequencies estimated in the E-step were the complete data as in method 3 except that the area-level exposure probabilities *X*_1*k *_and *X*_2*k *_(*k *= 1, 2, ..., 12) are not estimated from observed second-phase data but given externally from a GIS-database as a 3 × 12 probability matrix (additional file 3). These external probabilities are used together with observed frequencies for smoking in the E-step, when estimating the total frequency distribution with respect to the exposure and smoking categories across the areas for the non-participating subjects.

All methods are compared with an ideal situation in which individual-level information is available on all subjects. Data are assumed to be missing at random within each area, i.e. the participation rates in the second-phase interviews conditioned on disease status are not related to exposure. However, participation rates may differ between cases and controls and may also vary across the geographical areas, allthough in this study we do not incorporate any such differences in participation rates [17]. All methods are expected to produce bias-free estimates in the current study scenarios.

The 95% confidence intervals (CIs) around the estimates were calculated in order to appreciate the coverage of the methods. When using second-phase data only, confidence intervals of the Wald type were calculated [26]. The variance (for calculating the confidence intervals) in method 2 were estimated according to additional file 2. When estimating the effect with the EM algorithm (i.e. methods 3 and 4), the log-likelihoods including estimated expected exposure data as though they were observed cannot be used to estimate standard errors correctly [16], and therefore the confidence intervals of Wald type would not yield correct coverage. The standard errors were therefore estimated with the ideal bootstrap technique [27].

## Results

Unbiased or marginally biased (<7%) OR estimates were obtained with all methods (tables 3 and 4). The positive bias induced if smoking is not taken into account is approximately 20% for OR = 1.5, and 33% for OR = 3. The bootstrap technique yielded 95% CIs with accurate coverage and balance for methods 3 and 4; also for the other methods, the coverage was around 95% (data not shown).

Comparing method 3 with method 4 in table 3, the efficiency of method 4 is substantially higher, especially in scenarios 1 and 3. The results obtained with methods 1 and 2 have substantially lower efficiencies than the efficiencies obtained with method 4 in scenarios 1 and 3. In scenario 2, all methods generally perform more equally regarding efficiency, although method 4 still performs better than the others.

In table 4, the efficiencies both for methods 3 and method 4 are generally low and rather equal, although generally, method 4 has higher efficiency than method 3.

To sum up, the gain in efficiency is generally larger when incorporating external area-level data (method 4) than when calculating the area-level probabilities from the individual-level data (method 3).

## Discussion

We have simulated a specific study situation with three exposure categories and a binary confounding factor where additional data collection is desired due for example to missing-data, participation issues, or cost limitations. The simulated study resembles the data we will have access to in our planned case-control study regarding long-term exposure to air pollution and the risk of stroke with smoking as a potential confounder and effect modifier. Generalizations to polytomous exposure categories and confounding factors are straightforward.

We evaluated which of methods 1–4 that performs best with respect to efficiency and precision. Also, we evaluated how the methods perform when varying the number of cases and controls in the first and second phase, to get the optimal design for our planned case-control study. Our simulations compared the efficiencies of methods 1–4 relative to an ideal situation where individual-level information on all variables is available in the first phase. The simulated scenarios were such that all methods produced practically bias-free estimates. Our results indicates that in our study setting, the efficiency is greater with method 4 than with the other two-phase methods (methods 2 and 3) and, as anticipated, greater than the efficiency of method 1 where only second-phase data are used. Method 4 performed better than the other methods in all scenarios (tables 3 and 4). Regarding the optimal design for our planned study, for method 4, there does not seem to be much to gain in precision of the effect estimates by including a larger number of first-phase subjects than in scenario 1. In practice, method 4 is well suited to include many controls in the first phase, due to its use of register data, but we can conclude that the empiric standard errors of method 4 is rather constant when increasing the number of first-phase subjects compared to the initial scenario 1.

In order to gain efficiency compared to the ideal situation, it seems more important to include a large proportion of the first-phase cases than of the first-phase controls into the second phase for method 4, as seen in table 4, where scenario 2.2 has the lowest standard deviations. For method 3, the efficiencies in table 4 are rather equal in all scenarios.

The efficiencies obtained with method 2 in our setting are substantially lower than the efficiencies obtained with method 4, and sometimes even lower than the ones obtained with the second-phase method, method 1 (table 3). This is because method 2 uses second-phase data only for confounding adjustment. Method 2 has been used previously where individual-level data either on exposure or confounding factors are available in the first phase, and it turned out to be an efficient method [28]. However, the variance for the unadjusted first-phase estimate (equation 1) is relatively higher in our present study setting because we use area-level, and not individual-level, first-phase data. In our setting, participation bias is not present, but generally, method 2 is still preferable to method 1, since method 1 does not adjust for potential bias to the second phase.

We only considered scenarios where no participation bias within areas was present. If this assumption cannot be met, neither of the methods would be expected to produce bias-free estimates [17]. Both method 3 and 4 allow for the missing-completely-at-random assumption to be violated if the selective participation is between areas, but not if it is within areas. For example, if the control participation decreases in areas where a large proportion of the population is exposed to high levels of air pollution, this would not per se generate any bias in the estimates of methods 3 and 4. If there is participation bias within an area, for example if participation is related to individual disease status and exposure status, method 3 and 4 may both yield bias. If the area-level exposure data obtained externally are incorrect, methods 2 and 4 would not be expected to produce bias-free estimates. Even if method 4 gains slightly in precision with an increasing number of cases relative to the number of controls in the second phase, highly unbalanced designs would substantially enforce any bias stemming from erroneous exposure data. We only stratified the sampling in the second phase on case/control-status. In practice, the efficiency of the study would increase if we sample larger number of subjects in areas where there is a contrast in exposure distribution compared to areas where the individual-level exposure tend to be more equal. Also, depending on how the exposure is distributed in the population it might be desirable to over-sample subjects from areas with a small population rather than recruiting subjects in exact proportion to the population; for more about sampling in a two-phase design, see [29,30].

We stress that participation bias in population-based case-control studies is of great concern [31]. Analysis of the data with two-phase methods can reduce the bias and at the same time improve efficiency [14,17]. Two-phase designs are attractive in partially ecological studies where register data are available and additional data must be collected, a situation in which method 4 can be more effective than method 3. The proposed set-up could be generalized to any situation where individual-level register data are available on disease status and general covariates, and where group affiliation (e.g. residential area) allows for exposure to be assessed at the ecological level, using GIS for example, and where more exact exposure assessment and information on potential covariates for which no register data is available can be gathered in the second phase.

Several studies has been using distance to road as a proxy variable for air pollution [9,10,32]. The proportion in an area living within a certain distance to high-traffic roads could be used as first-phase variable with method 4.

In this paper, we have focused on studying long-term health effects of air pollution but there is also evidence of short-term health effects of air pollution [6]. Two-phase designs could be well-suited also for the study of acute effects of air pollution. The first-phase data could, for example, be the proportion of days in which a certain level of air pollution in different residential areas is exceeded, and the second-phase information could involve finding out the location of the study subjects at the time of the acute illness.

Previous studies have shown that the incidence of stroke may be associated with socio-economic factors [33,34], and that socio-economy can be an effect modifier for the association between air pollution and stroke [35]. When access to data is limited to ecological data, and no individual-level data is available, it may not be possible to differentiate between contextual and compositional effects [36]. Such a differentiation between effects can be enabled in a two-phase design. In our study on air pollution and stroke, we intend to adjust for compositional effects of socio-economic level in a two-phase design. Contextual variables that could be constructed through aggregation include average income, proportion unemployed or other socio-economic characteristics in the neighbourhood. Both methods 3 and 4 could be used to differentiate contextual and individual effects from each other. For this purpose, method 4 can be valuable in settings in which individual-level information is sparse but where ecological information is available, by incorporating the ecological information in the case-control analysis. Such extensions will require more complex statistical modelling, incorporating also random effects.

## Conclusion

We have illustrated the differences in performance between three two-phase methods in a study situation where exposure is only available at the area-level in the first phase and where individual-level exposure data is collected in the second-phase. The method that incorporates area-level exposure data in the first-phase, supplemented with individual ascertainment of exposure and confounders for a subsample, yielded the highest efficiency.

## List of abbreviations

Expectation-Maximization (EM)

Geographical Information Systems (GIS)

Maximum-Likelihood (ML)

Nitrogen dioxide (NO_2_)

Odds ratio (OR)

## Competing interests

The author(s) declare that they have no competing interests.

## Authors' contributions

AO: Contributed to study plan and methodological developments. Conducted the simulation study. Responsible for writing the manuscript.

JB: Contributed to study plan, methodological developments and the manuscript writing.

US: Contributed to study plan, methodological developments and the manuscript writing.

## Supplementary Material

Additional File 1 Overview of two-phase methods. We provide a more detailed description of the two-phase concept and a brief history of the development of two-phase methods Click here for file

Additional File 2 Appendix 1. A two-phase method for confounding adjustment in multiplicative models [25]. Click here for file

Additional File 3 Appendix 2. The EM method for method 3 and 4. Click here for file
