# Supplementary material for: Efficiency of two-phase methods with focus on a planned population-based case-control study on air pollution and stroke
Source: Environ Health. 2007 Nov 7;6:34. doi: 10.1186/1476-069X-6-34 (PMC2174445; doi:10.1186/1476-069X-6-34)
Supplement: Additional File 1 — Overview of two-phase methods. We provide a more detailed description of the two-phase concept and a brief history of the development of two-phase methods [file 1476-069X-6-34-S1.rtf]

Overview of two-phase methods
The first-phase variables available in a two-phase case-control study are disease status, and often residential area and general covariates like age and sex. It is also possible that the first phase provides some information on exposure at the individual or area (ecological group) level and on confounding factors (for example, socio-economic status). The sampling of the second-phase subjects in a case-control study for the collection of the second-phase data can depend both on disease status and other first-phase variables. Second-phase data collection can either be on variables other than those that are available in the first-phase (for example, confounder assessment) or on improved assessment of crude or error-prone first-phase variables. Two-phase studies are often more efficient than traditional designs and may account for bias due to varying participation rates, or varying sampling fractions, across areas. If participation bias is present within areas, however, a two-phase design can not be used to account for that type of bias.
In practice, a common approach among epidemiologists is to analyze the data from the second-phase subjects only, and to ignore first-phase subjects with missing individual-level data on exposure. However, that approach can lead to bias and a large loss of efficiency [1, 2]. It is feasible to adjust for participation bias or selection bias due to design and to reduce the standard errors of the parameter estimates to an adequate extent by incorporating the data from the first-phase-only subjects (i.e., subjects that were not sampled for, or did not participate in the second phase) into the data analysis. 
White [3] described such a two-phase analysis for a case-control study where a dichotomous exposure and disease status is known for all subjects but the evaluation of a covariate is desired by selecting a sub-sample of the subjects. For multiplicative models, Cain and Breslow [1] generalized the method described by White to include both multiple exposure categories and several covariates. This was done by using a pseudo maximum-likelihood approach estimating parameters in a two-phase design. The formulae for the necessary adjustments are fairly easy; see Appendix 1. For assessment of non-multiplicative joint effects between first- and second-phase variables, however, the method proposed by Cain and Breslow cannot be directly generalized.
Dempster et al. [4] introduced the iterative expectation-maximization (EM) algorithm, for computation of maximum likelihood (ML) estimates in missing data situations. Wacholder and Weinberg [5] proposed the EM algorithm for effect estimation in two-phase case-control studies. Their approach yield valid joint effect estimates between the first- and second-phase variables under both multiplicative and non-multiplicative models. 
Exposure information on group level is frequently available in occupational/environmental epidemiology. Two-phase analysis can be used to increase precision and to reduce bias in settings where data in the first phase are partially or purely ecological [2, 6]. Jackson et al obtained ML estimates for a combined model of area-level data on disease status , exposure, and covariables in the first phase and individual data on these variables for a subsample in the second phase [6].
Strömberg and Björk [2] used the EM algorithm in order to obtain ML estimates from the additive-multiplicative regression model for the exposure-disease association on the individual-level in a situation where disease status, group affiliation (e.g. occupational group or residential area), and general covariates such as age, and sex were known individually for all subjects in the first-phase. The association between the first-phase exposure variable, given by population estimates of the exposure probability in each group/area  and the individual disease risk could be assumed to be linear conditional on other covariates in the model [7].Second-phase data provided individual-level exposure data, which were assumed to be dichotomous, but no further covariates were taken into account. Note that Strömberg and Björk considered a study situation somewhat different from the two-phase designs described by, for example, Wacholder and Weinberg [5] and McNamee [8]. In the general two-phase sampling scheme, data are collected on individual level only. By contrast, Strömberg and Björk, as well as Jackson et al [6] collected individual-level data in the second phase on an exposure variable that was available at the ecologic level (as exposure probabilities) in the first phase. Whenever available, the second-phase (individual-level) exposure data replace the first-phase (ecologic-level) exposure data in the analysis by Strömberg and Björk. To obtain bias-free estimates with such replacements, however, no contextual effects of area affiliation or ecologic-level variables should be present.  If contextual effects are present, a mixed model allowing for residual random effect within areas would be more appropriate [9].


1.	Cain K, Breslow N: Logistic regression analysis and efficient design for two-stage studies. American Journal of Epidemiology 1988, 128:1198-1206.
2.	Strömberg U, Björk J: Incorporating group-level exposure information in case-control studies with missing data on dichotomous exposures. Epidemiology 2004, Jul;15(4):494-503.
3.	White JE: A two stage design for the study of the relationship between a rare exposure and a rare disease. Am J Epidemiol 1982, 115(1):119-128.
4.	Dempster A, Laird N, Rubin D: Maximum Likelihood from Incomplete Data via the EM Algorithm. Journal of the Royal Statistical Society 1977, 39(1).
5.	Wacholder S, Weinberg C: Flexible Maximum Likelihood Methods for Assessing Joint Effects in Case- Control Studies with Complex Sampling. Biometrics 1994, 50:350-357.
6.	Jackson C, Best N, Richardson S: Improving ecological inference using individual-level data. Statistics in Medicine 2006, 25(12):2136-2159.
7.	Bouyer J, Hemon D: Comparison of Three Methods of Establishing Odds Ratios from a Job Exposure Matrix in Occupational Case-Control Studies. Am J Epidemiol 1993, 137(4):472-481.
8.	McNamee R: Optimal design and efficiency of two-phase case-control studies with error-prone and error-free exposure measures. Biostat 2005, 6(4):590-603.
9.	Wong G, Mason W: The Hierarchical Logistic Regression Model for Multilevel Analysis Journal of the American Statistical Association 1985, Vol. 80(No. 391 (Sep., 1985)):513-524.
