# Supplementary material for: Efficiency of two-phase methods with focus on a planned population-based case-control study on air pollution and stroke
Source: Environ Health. 2007 Nov 7;6:34. doi: 10.1186/1476-069X-6-34 (PMC2174445; doi:10.1186/1476-069X-6-34)
Supplement: Additional File 2 — Appendix 1. A two-phase method for confounding adjustment in multiplicative models [25]. [file 1476-069X-6-34-S2.rtf]

Appendix 1 
Two-phase method for confounding adjustment in multiplicative models (Cain and Breslow [1] )
For multiplicative risk models, the log-odds ratio  for the combined first- and second-phase data (1+2) for exposure category i (i > 0) compared with the reference exposure category (i = 0) can be estimated with adjustments for confounders in the following way [26]:
	           
In our setting, smoking is a potential confounding variable when the association between air pollution and stroke is investigated. Air pollution exposure is assessed for all study subjects, but smoking status is only assessed for the second-phase subjects. The  is the estimated air pollution-effect odds ratio obtained with logistic regression when analyzing the second-phase data only, corrected for the smoking status (and, possibly, other covariates with data collected for the second-phase subjects). The is the effect estimate based on first-phase data only, thus unadjusted for the smoking status and other potential second-phase covariates, and  is the corresponding unadjusted estimate based on second-phase data. 

1.	Cain K, Breslow N: Logistic regression analysis and efficient design for two-stage studies. American Journal of Epidemiology 1988, 128:1198-1206.
