# Supplementary material for: Efficiency of two-phase methods with focus on a planned population-based case-control study on air pollution and stroke
Source: Environ Health. 2007 Nov 7;6:34. doi: 10.1186/1476-069X-6-34 (PMC2174445; doi:10.1186/1476-069X-6-34)
Supplement: Additional File 3 — Appendix 2. The EM method for method 3 and 4. [file 1476-069X-6-34-S3.rtf]

Appendix 2 
The EM algorithm for method 3 and 4
The number of subjects  with disease status D = d in area A = k and with missing second-phase data is defined as , where  are the first-phase subjects and  are the observed second-phase subjects. The second-phase vector of covariates S has two dimensions (S1 S2) in our setting: S1 = i denotes exposure category (0 = Low, 1 = Medium, 2 = High) and S2 = j denotes smoking category (0= Non-smoker, 1 = Smoker). In the (iterative) EM algorithm, we denote the probability of disease status D = d, based on the current estimates from the risk model on the individual level, as , where S is indexed as . Note that 
	 = , 
since area A is not part of the risk model on the individual level. However, we allow for varying participation rates across areas and we therefore estimate the frequency distribution of the missing second-phase data for each area separately. Thus, we can estimate the total (observed+expected) number of subjects  with disease status D = d, second-phase stratum S = s, in area A = k as [1]:
	.	(A1)

Method 3. We apply Bayes' theorem to formulate  as
,
where , is the total number of subjects (cases plus controls) in area A = k, second-phase stratum S = s, estimated in the previous iteration of the algorithm and . 

Method 4. Here,  in (A1) is formulated as 
	
	.	(A2)
For controls (D = 0),  is retrieved externally from the GIS-database. The corresponding probabilities for cases (D = 1) are obtained from the following system of equations:
	 (i > 0)
	,
where  is the current estimate for exposure category S1 = i versus the reference category (S1 = 0).
Solving the equation system for   yields the following expression:


Furthermore, we apply Bayes' theorem to express  in (A2) as
	
	
where , is the total number of subjects in area A = k, second-phase stratum S = s (S1 = i, S2 = j),  is the total number of subjects in area A = k, exposure category S1= i, both estimated in the previous iteration of the algorithm. 

Iterations. In the initial step of the algorithm in both method 3 and 4, maximum likelihood estimates of the risk model on the individual-level are obtained based on observed second-phase data only. In all subsequent steps, maximum likelihood estimates are obtained based on the sum of the observed and expected second-phase frequencies (A1), as if they were all observed. The procedure is repeated until convergence.


1.	Wacholder S, Weinberg C: Flexible Maximum Likelihood Methods for Assessing Joint Effects in Case- Control Studies with Complex Sampling. Biometrics 1994, 50:350-357.
